# The Role of Calcium Signaling in Melanoma

**DOI:** 10.3390/ijms23031010

**Published:** 2022-01-18

**Authors:** Haoran Zhang, Zhe Chen, Aijun Zhang, Anisha A. Gupte, Dale J. Hamilton

**Affiliations:** 1 Center for Bioenergetics, Houston Methodist Research Institute, Houston, TX 77030, USA; diudiutianmi@126.com (H.Z.); azhang@houstonmethodist.org (A.Z.); aagupte@houstonmethodist.org (A.A.G.); 2 Xiangya Hospital, Central South University, Changsha 410008, China; zchen347@163.com; 3 Department of Medicine, Houston Methodist, Weill Cornell Medicine Affiliate, Houston, TX 77030, USA

**Keywords:** calcium, melanoma, progression, melanoma microenvironment, mitochondria

## Abstract

Calcium signaling plays important roles in physiological and pathological conditions, including cutaneous melanoma, the most lethal type of skin cancer. Intracellular calcium concentration ([Ca^2+^]i), cell membrane calcium channels, calcium related proteins (S100 family, E-cadherin, and calpain), and Wnt/Ca^2+^ pathways are related to melanogenesis and melanoma tumorigenesis and progression. Calcium signaling influences the melanoma microenvironment, including immune cells, extracellular matrix (ECM), the vascular network, and chemical and physical surroundings. Other ionic channels, such as sodium and potassium channels, are engaged in calcium-mediated pathways in melanoma. Calcium signaling serves as a promising pharmacological target in melanoma treatment, and its dysregulation might serve as a marker for melanoma prediction. We documented calcium-dependent endoplasmic reticulum (ER) stress and mitochondria dysfunction, by targeting calcium channels and influencing [Ca^2+^]i and calcium homeostasis, and attenuated drug resistance in melanoma management.

## 1. Introduction

Cutaneous melanoma, one of the most malignant skin cancers, emerges from pigmented melanocytes. Although melanoma accounts for <2% of malignant skin tumors, it is the most aggressive form of skin cancer [1]. Calcium is a messenger molecule that plays several important roles in different physiological and pathological functions in cells, including melanoma cells. Calcium channels are widely expressed on several biological membranes, such as the mitochondrial, endoplasmic reticulum (ER), and plasma membranes. These channels regulate calcium flux and concentration under normal physiological conditions.

The calcium entry channels can be divided into (but are not limited to) receptor-operated calcium channels (ROCCs), voltage-dependent calcium channels (VDCCs), and store-operated calcium entry (SOCE) on the plasma membrane [2]. Glutamate receptor-mediated calcium channels, such as α-amino-3-hydroxy-5-methyl-4-isoxazolepropionic acid (AMPA), *N*-methyl-d-aspartate receptor (NMDAR), and metabotropic glutamate receptors (mGluRs), are ROCCs and have been widely studied in nerve cells [3]. However, their roles have been described in other cell types, including melanoma. It has been documented that blocking the NMDA receptor inhibits melanoma proliferation [4]. Furthermore, mGluR1 and mGluR5 expression is related to melanoma development [5,6]. VDCCs are located on the plasma membrane and are activated by electrical potential changes across the membrane. VDCCs can be classified into T-, L-, N-, R-, and P-/Q-subtypes, the expression of which varies among different cell types. Melanoma and melanocytes express high voltage-activated Ca(v)1 (L-types) and Ca(v)2 channels (N, P/Q, or R-types), while low voltage-activated Ca(v)3 channels (T-type) only exist in melanoma [7]. The depletion of Ca^2+^ is detected by the ER membrane protein STIM, which activates SOCE on the plasma membrane (Orai channels) and transient receptor potential calcium channels, including transient receptor potential melastatin (TRPM), transient receptor potential vanilloid (TRPV), and transient receptor potential canonical (TRPC), to allow Ca^2+^ influx [8,9]. Calcium efflux is supported by Ca^2+^-ATPase pump and sodium calcium exchanger (NCX); the latter is not only in the plasma membrane but in the mitochondria and ER membrane.

The ER is one of the largest membrane-bound cellular calcium storage organelles. The ER transmembrane ryanodine receptor (RyR) and inositol 1,4,5-trisphosphate receptor (IP_3_R) channels mediate calcium release from the ER into the cytosol. Sarco/endoplasmic reticulum Ca^2+^-ATPase (SERCA) facilitates calcium pumping from the cytosol into the ER in an ATPase-dependent manner [10].

**Figure 1:**
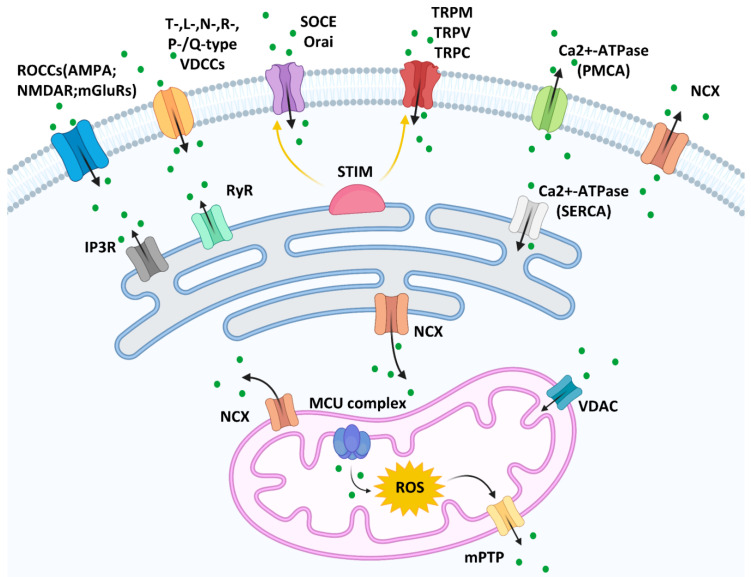
Calcium channels on the membranes of plasma, mitochondria, and the endoplasmic reticulum [14].

Mitochondria also play an important role in calcium homeostasis. Voltage-dependent anion channels (VDACs) located in the outer mitochondrial membrane increase the Ca^2+^ uptake into the intermembrane space [11]. Mitochondrial calcium uniporter (MCU) complex, which contains the major protein MCU and regulatory subunits MICU1/2, EMRE, and MCUb, is the major mediator of mitochondrial Ca^2+^ uptake on the mitochondrial inner membrane [12]. High matrix concentrations of Ca^2+^ with reactive oxygen species (ROS) in the mitochondria trigger mitochondrial permeability transition pore (mPTP) opening and release the Ca^2+^ into the cytoplasm [13]. (Figure 1).

## 2. Calcium Signaling in Melanogenesis and Melanoma Tumorigenesis

### 2.1. Calcium Signaling in Melanogenesis

Calcium signaling plays a pivotal role in melanogenesis, which has effects on melanoma tumorigenesis and therapeutic outcomes [15]. Pigmentation can be regulated by membrane voltage changes mediated by modulating calcium channels with increased cytosolic Ca^2+^ influx. Cytosolic Ca^2+^ transports into melanosomes to increase tyrosinase activity, trigger melanin transfer, or regulate organelle interaction by activating PKCβ [16]. For example, repression of TRPM1 expression results in reduced intracellular Ca^2+^ and decreased uptake of extracellular Ca^2+^, accompanied by attenuated activity of the melanogenic enzyme tyrosinase and melanin pigment [17]. Sun et al. found that the stimulation of melanogenesis by synaptotagmin-4 is mediated by regulating Ca^2+^ influx through TRPM1 [18]. In addition, the release of internal Ca^2+^ stores through the Orai/STIM pathway increases tyrosinase activity and melanin content; this is triggered by solar ultraviolet radiation-induced endothelin-1 release [19]. Two-pore channel (TPC) located on the lysosomes, late endosomes, and melanosomes was reported to increase the risk of developing skin cancer by decreasing the melanin production and regulating melanosome maturation modulated by mTOR [16,20].

### 2.2. Calcium Signaling in Melanoma Tumorigenesis

**Figure 2:**
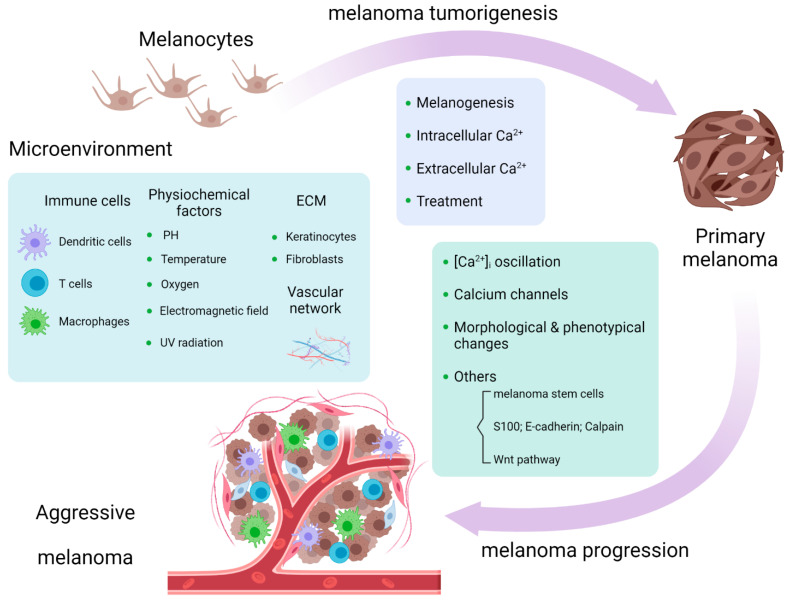
Calcium signaling is involved in melanoma tumorigenesis and progression and melanoma microenvironment [58].

Melanoma tumorigenesis is a process whereby a benign melanocyte transforms into a primary melanoma in which the calcium influx across multiple cellular compartments is a key controller of the process [21]. Aside from the role in melanogenesis, calcium-related pathways are involved in the tumorigenesis of melanoma. IP3-mediated Ca^2+^ release from intracellular stores activates non-phosphorylated PKC isoforms which act as tumor promoters and are linked to carcinogenesis; some isoforms especially, PKC α and β, represent a malignant phenotype in melanoma [22,23]. PAR1 signaling accelerates calcium mobilization. The downstream pathways of PAR1 signaling, such as the activation of MAPKs, are involved in melanoma tumorigenesis [24]. Although there is no direct evidence that PAR1-induced Ca^2+^ flux affects melanoma tumorigenesis, it is worthy of further investigations. Extracellular Ca^2+^ regulators play important roles in melanoma tumorigenesis. Robert et al. documented that the extracellular Ca^2+^-binding matricellular glycoprotein SPARC promotes early transformation of melanocytes by mediating E-cadherin suppression and Snail induction [25]. PRP4 blocks the Ca^2+^ influx through desensitization of the extracellular calcium sensing receptor (CaSR), with the involvement of TRP cation channel subfamily C member 1, which is the promoting factor of skin carcinogenesis [26]. Altering Ca^2+^ homeostasis by targeting lipid rafts, the cholesterol-enriched membrane microdomains in melanoma cells, abolishes activated PKB, rendering melanoma susceptible to apoptosis and attenuating its tumorigenicity; this can act as a therapeutic target in melanoma prevention [27]. From the glimpse of the role of calcium signaling in melanoma tumorigenesis, we conclude that calcium flux controls melanoma tumorigenesis mainly through calcium-related pathways, which requires further investigations about the direct impact of Ca^2+^ on the carcinogenesis of melanoma. (Figure 2).

## 3. Calcium Signaling in Melanoma Progression

Melanoma progression happens when the primary melanoma progresses to a metastatic melanoma with a migrating and invading capacity. Intracellular calcium concentration ([Ca^2+^]i) and its multiple channels function as regulators of melanoma progression that serve as mechanistic targets for control of melanoma growth and management of metastasis.

### 3.1. [Ca^2+^]i Oscillation Influences Melanoma Progression

Evidence documents that increased intracellular calcium stores are associated with highly metastatic melanoma cells [28]. Calcium released from ER facilitates melanoma cell migration. Epac1 activated by cAMP induces calcium elevation from ER via the PLC/IP_3_ receptor pathway and facilitates cell migration with the involvement of actin assembly, which is inhibited by mSIRK, a Gβγ-activating peptide, activating calcium influx from the extracellular space [29,30]. The expression of cGMP phosphodiesterase PDE5A is downregulated by oncogenic BRAF in BRAF^V600E^ mutated melanoma by the extracellular-signal-regulated kinase (ERK) pathway, which induces an increase in [Ca^2+^]i, stimulating melanoma cell invasion and short-term and long-term lung colonization [31]. Y-box binding protein 1 is an unfavorable prognostic marker secreted from melanoma depending on [Ca^2+^]i and ATP levels, the expression of which increases in primary and metastatic melanoma, compared to benign melanocytic nevi. Conversely, elevated Y-box binding protein 1 secretion stimulates melanoma cell migration, invasion, and tumorigenicity [32]. Paradoxically, increased [Ca^2+^]i was reported to decrease melanoma progression. Olfactory receptor 51E2 activated by its ligand β-ionone suppresses the migration of vertical-growth phase melanoma cells by increasing [Ca^2^+]i [33].

### 3.2. Calcium Channels Are Involved in Melanoma Progression

Since [Ca^2+^]i plays an important mechanistic role in melanoma progression, the role of calcium channels cannot be neglected. Basically, NMDAR calcium channel function is weak in melanoma cells but strongly contributes to cell proliferation and invasion when its encoding gene GRIN2A is mutated at certain sites, such as G762E, with less glutamate supplementation [34]. Another glutamate receptor calcium channel mGluR5 was proved to have a profound effect on melanoma progression in vivo by triggering the phosphorylation of ERK [5]. The ERK pathway is also implicated in SOCE-mediated melanoma progression. Inhibition of SOCE by knockdown of STIM1 or Orai or by SOCE inhibitors suppresses melanoma cell proliferation and migration, while induction of SOCE activates ERK, which is inhibited by calmodulin kinase II or Raf-1 inhibitors [35]. TPC2 influences melanoma progression via SOCE. Downregulation of TPC2 expression in metastatic melanoma leads to a decrease of Orai1 expression and an increase of YAP/TAZ activity, which is responsible for melanoma’s aggressive property [36]. In BRAF mutant melanoma—the BRAF^V600E^ mutation in particular—the expression of Ca^2+^-ATPase isoform 4b (PMCA4b) on the plasma membrane is low compared with benign nevi and is markedly elevated by vemurafenib (BRAF inhibitor) or selumetinib (MEK inhibitor) treatment, which indicates crosstalk between PMCA4b and the MAPK pathway. Activation of p38 MAPK induces the degradation of PMCA4b, while suppression of p38 MAPK by increasing the abundance of PMCA4b promotes the [Ca^2+^]i clearance and inhibits the migration of melanoma cells [37,38]. Moreover, SERCA on the ER membrane, controlled by the interaction between calcium-modulating cyclophilin ligand and basigin, was reported to have an effect on invasion and metastasis by regulating [Ca^2+^]i and matrix metalloproteinase (MMP)-9 activity in A375 cells [39]. Unlike Ca^2+^-ATPase, T-type VDCCs drive migration and invasion in BRAF mutant melanoma cells depending on Snail1 levels, suggesting therapeutic strategies by blocking T-type VDCCs to inhibit progression of melanoma [40]. Other ion channels are implicated in melanoma progression through calcium signaling. Nav1.6 sodium channel promotes melanoma cell (WM266 and WM115) invasion and proliferation by mTOR-mediated Na^+^/Ca2^+^ exchange [41]. KCa3.1 potassium channel was reported to promote melanoma cell migration by controlling the secretion of melanoma inhibitory activity proteins depending on [Ca^2^+]i [42].

### 3.3. Ca^2+^ Signaling Influences Melanoma Progression through the Change of Morphological and Phenotypical Changes

Ca^2+^ signaling also leads to cell morphological and phenotypical changes, including the elongated cell axonal- and mesenchymal-like shape, formulation of invadopodia, and altered cytoskeleton structure, making cancer cells become more deformable and more invasive. Except for the role in melanogenesis, synaptotagmin-4 is thought to have a relationship with the growth and metastasis of melanoma by influencing axonal elongation [43]. Orai- and STIM1-mediated Ca^2+^ oscillation signals were reported to facilitate invadopodium assembly and thus promote melanoma invasion by regulating the recycling of membrane-bound MT1-MMP and extracellular matrix (ECM) remodeling [18,44]. The effect of the β2-adrenergic–Ca^2+^–actin axis on cancer invasion was reported in melanoma and other cancer types. β-adrenergic receptor (βAR) signaling triggers actin remodeling and reorganization to enhance cell contractility and promote cell invasion. β-adrenergic receptor-induced Ca^2+^ acts as a regulator of cytoskeletal actin by directly binding to actin or binding to filamin, the crosslinker of actin [45]. Meghnani et al. reported the upregulated expression of receptor for advanced glycation end products (RAGE) in melanoma patients in late metastatic stages. Overexpression of RAGE induced melanoma cells to become more metastatic by triggering cells into mesenchymal-like morphologies, which is associated with the upregulation of its ligand S100B, a calcium-binding protein [46].

### 3.4. Calcium-Related Pathways Participate in Melanoma Progression

Other factors (melanoma stem cells), other proteins (S100 family, E-cadherin, and calpain), and the Wnt/Ca^2+^ pathway influence melanoma progression through calcium signaling. Ca^2+^ released through IP_3_R in melanoma cells is crucial for the function of cancer stem cells. IP_3_R impairment leads to a diminution in the population of melanoma stem cells and reduced melanoma growth [47]. A network analysis of the expression of Ca^2+^ signaling and stem cell pluripotency-related genes (e.g., GSTP1, SMAD4, CTNNB1, MAPK3, GNAQ, PPP1CC, GSK3B, and PRKACA) showed some candidates that may contribute to the melanoma metastatic transformation and potential therapeutic biomarkers for metastatic melanoma [48].

S100A4 is a metastasis-promoting protein in melanoma cells which acts by targeting metabolic reprogramming, that is, the suppression of mitochondrial respiration and the activation of aerobic glycolysis [49]. Upregulation of S100P, ezrin, and RAGE improves the malignancy of melanoma [50]. E-cadherin has extracellular Ca^2+^-binding domains whose functions are dependent on Ca^2+^ and is essential for melanogenesis and melanoma suppression. E-cadherin silencing is related to melanoma metastatic dissemination and poor prognosis [51,52]. The decreasing expression of E-cadherin by overexpression of T-box transcription factors Tbx2 and Tbx3 is associated with enhanced melanoma invasiveness [53]. Promoter methylation by activating E-cadherin expression represents its therapeutic role in the treatment of melanoma [51]. Evidence in vitro and in vivo showed that inhibition of calpain, whose activity is promoted by calcium signaling, blunts melanoma growth, allows melanoma cells to escape from anti-tumor immunity, and increases metastatic dissemination by accelerating the migration process and reducing apoptosis [54].

Wnt5a was found to be expressed in highly aggressive melanoma and was able to increase melanoma invasive potential by activating PKC and raising [Ca^2+^]i in a transfected model [55]. Interestingly, Wnt5a signaling was engaged into melanoma cell movement, rendering them more aggressive. Wnt5a leads to the remodeling of the cytoskeleton and increases melanoma motility by activating calpain-1, leading to the cleavage of filamin A [56]. The assembly of the “Wnt-receptor-actin-myosin-polarity” structure, which is promoted by Wnt5a, promotes actomyosin contractility and substrate detachment for membrane retraction, mediated by the recruitment of cortical ER and elevation of Ca^2+^ [57]. (Figure 2).

## 4. Calcium Signaling in Melanoma Microenvironment

The tumor microenvironment, including surrounding immune cells and other cells, signaling molecules, blood vessels, and ECM, is closely related to and constantly interactive with melanoma cells, playing pivotal roles in melanoma generation, progress, and prognosis. Calcium signaling influences the altered microenvironment to change the fate of the melanoma by influencing the function of innate and adaptive immune cells, regulating ECM and tumor vascularization, and adapting to different physical and chemical surroundings.

### 4.1. Immune Cells

In T cell-based tumor immunosurveillance, cytotoxic T lymphocytes (CTLs) kill tumor cells by recognizing their specific T cell receptor. It was proved that CTLs-mediated cytotoxic function in melanoma and other cancers depends on a SOCE-mediated [Ca^2+^]I rise by regulating the degranulation of CTLs, the production of TNFα and IFNγ, and the expression of Fas ligand both in vivo and in vitro [59]. CD4^+^CD25^+^Foxp3^+^ regulatory T cells cause effector T cell death and suppress activation of T cells to induce immunosuppression through TGFβ-induced inhibition of IP_3_ production with a decrease in intracellular Ca^2+^ flux. Accordingly, Kim et al. increased IFNγ production and activated T cells in vitro and reduced melanoma growth in vivo through highly selective optical control of Ca^2+^ signaling in CTLs [60]. EGR4, a member of the zinc finger transcription factor family, was reported as a key regulator of T cell differentiation. Knocking out EGR4 in T cells triggers an enhanced Ca^2+^ response and increased IFNγ production in vitro and leads to regulatory T cells loss, Th1 bias, and CTL generation in a mouse melanoma lung colonization model [61]. Histamine and its H_4_ receptor induce the chemotaxis and migratory properties of γδ T cells through G_i_ protein-dependent [Ca^2+^]i increase in the microenvironment of melanoma cells [62].

Moreover, Ca^2+^ flux was involved in the NK cell-mediated innate immune response to melanoma cells. Although no difference in the formation of metastatic lung lesions was observed, NK cells are hyporesponsive to MHC class I-deficient target cells, with NK cells continuously activating by the Ly49H receptor [63]. Tumor-associated macrophages, especially CD163^+^ M2 macrophages, are related to immune escape, supporting cancer development [64]. Secreted flavoprotein renalase enhances the function of M2 macrophages to promote melanoma growth through the PMCA4b calcium channel by activating the MAPK and PI3K/AKT pathways [65]. Recently, mesencephalic astrocyte-derived neurotrophic factor, a novel immunoregulator basically secreted from pancreatic beta cells, was found to be secreted from melanoma and other cancer cell lines upon IFNγ-induced ER calcium depletion, which was proved to activate M2 macrophages and promote melanoma growth [66,67]. In addition, macrophages in the melanoma microenvironment are less susceptible to calcium electroporation compared with melanoma cells, but calcium electroporation stimulates the immunogenic capacity of melanoma-conditioned macrophages [68]. Calcium electroporation is a promising method in anti-cancer treatment under clinical trial which utilizes high-voltage electric pulses to introduce calcium flux into cells [69]. Recently, a near-infrared-stimulable optogenetic platform was established to remotely and selectively control Ca^2+^ oscillations and Ca^2+^-related gene expression and to modulate immunoinflammatory responses by regulating the functions of T lymphocytes, macrophages, and dendritic cells [70]. What is more, bone marrow-derived mast cells prefer to locate in hypoxic zones of the melanoma microenvironment, inducing CCL-2 synthesis and calcium rise by activating LVDCCs [71].

### 4.2. ECM and Vascular Network

In melanoma, ECM, molecules, proteins, and stromal cells interacting with Ca^2+^ signaling influence melanoma development. As we discussed above, Orai1- and STIM1-mediated Ca^2+^ oscillations regulate melanoma ECM degradation by MT1-MMP [18,44]. Attenuated [Ca^2+^]i enhances the chemotaxis of melanoma cells to type IV collagen, a member of the ECM proteins, depending on CD47 and integrins α_2_β_1_ and α_ν_β_3_ [72,73]. Thrombomodulin, an integral membrane glycoprotein on endothelial cells, acts as a Ca^2+^-dependent molecule controlling melanoma cell adhesion [74]. Kallikrein-related peptidase 6 is detected in neighboring stromal cells and keratinocytes and displays a paracrine function to accelerate melanoma migration and invasion which was proved to depend on protease-activated receptor 1-induced intracellular Ca^2+^ flux [24]. Skin keratinocytes and fibroblasts in melanoma ECM play important roles in melanoma development. Keratinocytes reduce the expression of TRPC1, 3, and 6 to decrease [Ca^2+^]i and negatively regulate the *N*-cadherin levels, a progressive factor in melanoma cells [75]. Keratinocytes can lead to cutaneous malignant lesions, dependent on the loss of calcium channel P2X_1–3_ and P2Y_2_ receptors and E-cadherin [76]. *N*-cadherin can promote melanoma cell migration and metastasis by facilitating the adhesion of melanoma cells to dermal fibroblasts and vascular endothelial cells [77].

The vascular network in the melanoma microenvironment, tightly interacting with ECM, provides nutrients and advantageous conditions for proliferation and metastasis. As we discussed above, the positive effects of Wnt5a on melanoma metastasis also include Ca^2+^-dependent exosome release, containing the pro-angiogenic and immunosuppressive factors (VEGF, IL-6, and MMP-2), which suppresses endothelial cell branching. Wnt5a expression has a potential relationship with the angiogenesis marker ESAM [78]. Nicotinic acid adenine dinucleotide phosphate, which is capable of triggering Ca^2+^ release from endosomes and lysosomes by targeting TPCs, was reported to control VEGF-induced angiogenesis in melanoma cells [79]. Moreover, vasculogenic mimicry is specific in less vascularized areas of the tumor microenvironment, providing nutrients and oxygen to facilitate tumor metastasis. Zhang et al. reported the role of the calcium/phospholipid-binding protein myoferlin in the inhibition of vasculogenic mimicry formation in melanoma by inducing mesenchymal-to-epithelial transition and decreasing MMP-2 expression [80]. The reconstitution of vascular mimicry with the combination of VEGFA signaling in ECM contributes to the formation of capillary-like structures in the melanoma microenvironment which is regulated by intracellular and extracellular Ca^2+^ levels and α_ν_β_3_ and α_ν_β_5_ integrins [81]. Studies displayed some anti-vascular methods in anti-tumor treatment by targeting Ca^2+^ signaling. Carboxyamido-triazole, an inhibitor of non-VDCCs, displayed inhibitory effects on melanoma invasion and angiogenesis, disrupting the signaling between melanoma and its microenvironment by suppressing VEGF production and endothelial cell response to VEGF [82]. Calcium electroporation not only directly induced melanoma necrosis and indirectly affected macrophages in the melanoma microenvironment but recently was found to suppress the formation of capillary-like structures in vitro and damage melanoma blood vessels in vivo [83,84]. Particularly, vascular endothelial cadherin is basically specific to endothelia but also presented in some melanomas [85]. Vascular endothelial cadherin-mediated interaction between melanoma and adjacent endothelium plays an important role in tumor metastasis properties. Inhibition of the PLC/IP_3_ pathway disrupts the melanoma–endothelium junctions by diminishing endothelial [Ca^2+^]i response [86,87].

### 4.3. Physical and Chemical Surroundings

The extracellular pH in melanoma is acidic because of the excess amount of anaerobic glucose metabolites [71]. Acidic extracellular pH enhances Ca^2+^ influx through VDCCs [88]. Noguchi et al. demonstrated therapeutic roles of mitochondrial inhibitors against melanoma accompanied by increasing [Ca^2+^]i at acidic extracellular pH, but a neutral or alkaline microenvironment enhanced melanoma growth and lung metastasis under the treatment of mitochondrial inhibitors [89]. Consequently, the tumor microenvironment was utilized to improve the treatment of melanoma. Cold atmospheric plasma induced Ca^2+^ influx in melanoma cells and acidification in the tumor microenvironment, which was thought to be the reason for its anti-cancer effects [90]. Except for low pH in the melanoma microenvironment, hypoxic conditions in melanoma lead to increased adenosine levels and high production of ROS [71]. Physical microenvironment changes, such as confinement, are able to elevate [Ca^2+^]i and suppress PKA activity via a PDE1-dependent pathway in melanoma cells which affects cell stiffness and locomotion [91]. Exposing melanoma cells to low-intensity, frequency-modulated electromagnetic fields for more than 15 min exhibits cytotoxic effects, with the involvement of VDCCs in an in vitro study [92]. Yu et al. reported the “cold/hot” properties of traditional Chinese medicine, which changes the temperature in A375 cells by TRPV4-mediated intracellular calcium influx [93]. UV radiation is a risk factor of melanoma. The roles of UV radiation in melanoma with calcium signaling involvement occur mainly by influencing vitamin D signaling, mitochondria-related Ca^2+^ influx, and ORAI1 channel-mediated melanogenesis [94,95,96] (Figure 2).

## 5. Calcium Signaling and Other Ionic Channels in Melanoma

### 5.1. Sodium Channels

Other ionic channels, including sodium and potassium channels, are engaged in the calcium transport systems. Normally, NCX transports three sodium ions into the cell and one calcium ion outside (forward mode), which can be performed in the opposite way (reverse mode) under special conditions [97]. Therefore, the function of sodium channels is relevant to the Ca^2+^ current. Nav 1.6, a voltage-gated sodium channel, is overexpressed in melanoma cells, compared with normal melanocytes. Inactivation of Nav 1.6 by its inhibitor tetrodotoxin suppresses aggressive properties and promotes apoptosis in melanoma cells by reducing mTOR activity and interrupting the translocation of mitochondrial Ca^2+^ flux [41]. Another study also evidenced the role of Nav 1.6 in regulating invasion by controlling the Ca^2+^-dependent podosome and invadopodium formation [98]. In melanoma cells with different metastatic capacities, Ca^2+^ buffering capacities are different. NCX functions in a reverse mode for Ca^2+^ entry, which leads to a sudden increase in [Ca^2+^]i in highly metastatic melanoma cells, while the NCX in lowly metastatic melanoma cells is in a forward mode, suggesting the vital role of NCX mode in melanoma metastasis characteristics [97,99]. Additionally, the expression of NCX1 varies between NRAS^Q61R^ and BRAF^V600E^ mutated human melanoma cells with different Ca^2+^ homeostasis and Ca^2+^-dependent aggressiveness. NRAS^Q61R^ mutated (SK-MEL-147) cells contain higher levels of NCX1 expression and exhibit more sensitivity to vemurafenib treatment with NCX inhibition as compared to BRAF^V600E^ mutated (SK-MEL-19) cells [100].

### 5.2. Potassium Channels

Ca^2+^-activated K^+^ (K_Ca_) channels can be divided into three subfamilies: small-conductance K^+^ (SK_Ca_) channels, intermediate-conductance K^+^ (IK_Ca_) channels, and big-conductance K^+^ (BK_Ca_) channels. Voltage-insensitive SK_Ca_ and IK_Ca_ channels are activated by low [Ca^2+^]i. In contrast, BK_Ca_ channels are activated by voltage and high [Ca^2^+]i [101]. K_Ca_ channels, especially the SK_Ca_ channels, are upregulated by hypoxia, which provides the underlying mechanism of enhanced proliferation in melanoma cells under hypoxic conditions [102]. KCa3.1 potassium channels, a subfamily of SK_Ca_/IK_Ca_ channels, were found to support the secretion of melanoma inhibitory activity, promoting melanoma cell migration [42]. The disruption of cholesterol rafts proximal to BK_Ca_ channels increases the activity of BK_Ca_ channels. In human melanoma IGR39 cells, Na^+^/K^+^-ATPase in the rafts that control intracellular Na^+^ levels was reported to influence the efficient functioning of BK_Ca_ channels [103]. Filamin A is also necessary for the normal function of BK_Ca_ channels, which normally traffic to the plasma membrane in A7 melanoma cells with filamin A but have trouble trafficking in M2 cells without filamin A [104]. Except for K_Ca_ channels, Ca^2+^-inactivated K^+^ channels were reported to control the proliferation of murine B16 melanoma cells, mediated by endothelin-1 [105].

## 6. Calcium Signaling in Melanoma Treatment

Taken together, calcium signaling is tightly related to melanogenesis, melanoma tumorigenesis and progression, and the melanoma microenvironment in consideration of its pivotal roles in melanoma growth. As we document above, multiple therapeutic strategies targeting calcium-related pathways were described during melanoma development from benign melanocyte to highly malignant melanoma, from melanoma itself to the surroundings. All in all, targeting calcium signaling in melanoma treatment is basically performed by targeting calcium channels and influencing [Ca^2+^]i and calcium homeostasis to directly kill melanoma cells or affect relative pathways. Here, we put emphasis on the strategies for melanoma treatment targeting Ca^2+^-related mitochondrial dysfunction and ER stress to illustrate those in consideration of the essential roles of ER and mitochondria in the regulation of calcium signaling. Additionally, calcium-related treatment can combine with other drugs in melanoma management by attenuating drug resistance in indirect manners.

### 6.1. Targeting Calcium, Mitochondria, and ER Stress in Melanoma

ER calcium imbalance can induce ER stress due to its capacity to accumulate unfolded proteins and, in turn, enhance Ca^2+^ efflux from the ER and feed mitochondrial Ca^2+^ uptake, triggering mitochondrial swelling, cell necrosis, and apoptosis. Specifically, mitochondrial Ca^2+^ overload triggers the formation of ROS, a decline in mitochondrial membrane potential, and opening of mPTPs with resultant release of the pro-apoptosis factor cytochrome c followed by activation of caspase-dependent and -independent apoptosis pathways [106,107].

Calcium channel dynamics are implicated in melanoma treatment targeting mitochondria/ER stress. Although SOCE-mediated Ca^2+^ responses are critical for melanoma proliferation and apoptosis [108], drugs with anti-melanoma effects, such as diallyl trisulfide [109], to induce mitochondrial Ca^2+^ overload, ROS production, and caspase activation are mediated not by SOCEs but VDCCs. Rouaud et al. reported ER stress in melanoma induced by a NADPH analog, NS1, relying on TRPM2 and Ca^2+^-activated K^+^ channels [110]. A combination of ER transmembrane protein selenoprotein K and ER enzyme DHHC6 can palmitoylate IP_3_R and stabilize Ca^2+^ flux. Impairing selenoprotein K promotes ER stress for melanoma progression [111,112]. In addition, mitochondrial Ca^2+^ overload contributes to the apoptosis-promoting effect of metformin and lectin, purified from Bothrops leucurus snake venom, on melanoma through mPTP opening [113,114]. Ribosomal protein S3 acts as a potential therapeutic target for melanoma treatment on account of its regulatory effects on mitochondrial Ca^2+^ and cascading apoptosis by mPTP and MICU1 [115]. Except for calcium signaling-related apoptosis, Raimondi et al. revealed that δ- tocotrienol triggered paraptosis, the nonapoptotic programmed cell death, caused by Ca^2+^ overload and ROS-associated mitochondrial dysfunction in melanoma cells. Additionally, δ-tocotrienol treatment also reduced mitochondrial membrane potential, oxygen consumption, and the expression of mitochondrial complex I. The mitochondrial Ca^2+^ overload was thought to be mediated by IP_3_R and VDAC [116].

Since calcium homeostasis is pivotal in ER stress and mitochondria-mediated cell death, several studies applied Ca^2+^-induced cell death to cancer treatment, including melanoma treatments such as luteolin, N-acetyl-S-(p-chlorophenylcarbamoyl) cysteine (NACC), and sanguinarine [107,117,118], which mechanically revealed their potential pathways. In particular, aripiprazole is not only an antipsychotic drug but a compound capable of depleting ER calcium in melanoma, thereby leading to activation of the unfolded protein response via protein kinase R-like ER kinase (PERK) and inositol-requiring enzyme 1 [119]. Another study found that the anti-tumor effects of polyphenols was also mediated by PERK-directed Ca^2+^ release [120]. Some molecules that are cytotoxic to melanoma cells, for example, digitoxin and MEK inhibitors, alter mitochondrial membrane potential and trigger mitochondrial calcium dysregulation, intracellular acidification, and ATP depletion by disrupting ion gradients and reducing ERK phosphorylation, respectively [121]. Imiquimod, a toll-like receptor (TLR) agonist, was demonstrated to induce ER stress and Ca^2+^ depletion followed by mitochondrial membrane potential loss and cytochrome c release, independently of TLR7 and TLR8, to trigger the apoptosis of melanoma cells, which was associated with Kinase 1/c-Jun-N-terminal kinase/p38 pathways. Apoptosis protein antagonists and NF-κB inhibitors can improve the effectiveness of imiquimod in melanoma treatment [122,123]. The underlying mechanism is the reduction of SOCE and mitochondrial Ca^2+^ loading as well as fragmentation, clustering, and swelling in mitochondria [124]. Recently, a study demonstrated that pulsed focused ultrasound induced DNA damage in melanoma cells by superoxide and H_2_O_2_ formation caused by Ca^2+^ homeostasis change [125]. Interestingly, photodynamic therapy can directly kill melanoma cells by triggering Ca^2+^-related ROS formation; it was proved to have “bystander effects” on nearby cells that are not exposed to light. Ca^2+^ released from the ER in a single exposed melanoma cell is capable of promoting mitochondrial O_2_^−^. formation in its bystander cells [126].

### 6.2. Drug Resistance and Combination Treatment

In some conditions, targeting calcium signaling is able to render melanomas more susceptible to conventional therapy, preventing the development of drug resistance and providing novel ideas for combination treatment. Molecular targeting T-type VDCCs is a promising solution for melanoma chemoresistance, since the Ca(v)3.1 isoform is high expressed in vemurafenib-resistant BRAF^V600E^ mutated melanoma. Mibefradil, a T-type VDCCs blocker, can restore the sensitivity of de-differentiated murine melanoma cells to MAPK inhibitors [127,128] and can reduce the motility and invasion capacity of BRAF^V600E^ mutants [40]. Silencing of Ca(v)3.1 or Ca(v)3.2 reduced the invasiveness of melanoma cells with BRAF^V600E^ mutation [129]. Tumor necrosis factor-related apoptosis-inducing ligand (TRAIL) is a promising anticancer drug, while some melanomas are resistant to TRAIL treatment. Studies demonstrated that Ca^2+^ dynamics are a promising approach to overcome TRAIL resistance. Mitochondrial Ca^2+^ removal increases TRAIL efficacy against melanoma through mitochondrial hyperfusion [130]. Interestingly, mitochondrial Ca^2+^ overload results in selective sensitization to TRAIL cytotoxicity by increasing mitochondrial fragmentation [131]. Cold plasma-stimulated medium exhibits its tumor-selective cytotoxicity in the treatment of TRAIL-resistant melanoma cells, as evidenced by mitochondrial network abnormalities, disrupting Ca^2+^ homeostasis and caspase-independent cell death [132]. Co-treatment with autophagy inhibitors and TRAIL displays promising therapeutic effects. NCX inhibitors altering Ca^2+^ flux sensitize NRAS^Q61R^ mutated melanoma cells to vemurafenib [96]. K_2_[B_3_O_3_F_4_OH] exhibits its cytotoxic effects on melanoma cells but not melanocytes only under low Ca^2+^ concentrations, suggesting the therapeutic effects of the combination of K_2_[B_3_O_3_F_4_OH] and methods for Ca^2+^ depletion [133]. Therefore, with the developing perception of calcium signaling in melanoma, it will provide more options for melanoma treatments and expand the pharmacological arsenal in the future.

### 6.3. S100 Protein Family in Melanoma Prediction

In addition to its therapeutic roles, calcium signaling is presenting potential diagnostic biomarkers for melanoma. We mainly document the role of the S100 protein family in the diagnosis of melanoma and prediction of prognosis. The S100 protein family, consisting of a Ca^2+^-binding EF-hand structure, is an important biomarker in serum that has been well studied in melanoma. S100B levels reflect the stage and prognosis in melanoma, due to its stage-dependent secretion [134]; in particular, it is considered as a biomarker of tumor load and progression in stage IV melanoma patients [135]. During the first week of anti-PD-1 therapy, S100B levels can also serve as a biomarker to predict the overall survival and response to the treatment and help to guide treatment decisions [136]. Other clinical studies revealed its predictive function in melanoma patients with BRAF inhibitor or CTLA-4 inhibitor treatment [137,138]. Nordlinger et al. proved that poor patient prognoses are correlated with high S100A4 expression levels [139]. In addition, high serum levels of heterodimer S100A8/S100A9 in early stages of melanoma patients with ipilimumab treatment predict worse response [1]. S100A13 is upregulated in melanoma, cooperating with VEGFA in supporting angiogenesis, leading the shift from radial to vertical growth [140]. Moreover, other calcium-associated biomarkers are being studied as well. For instance, a cross-sectional study showed that high levels of albumin-corrected serum calcium may predict the progression of malignant melanoma [141]. The expression of T-type VDCCs is increased in BRAF^V600E^ mutated cells, especially in those resistant to MAPK inhibitors, and this can serve as valuable prognostic markers in melanoma [129].

## Data Availability

Not applicable.
